# Management, morphological and genetic diversity of domesticated agaves in Michoacán, México

**DOI:** 10.1186/s13002-020-0353-9

**Published:** 2020-01-16

**Authors:** Gonzalo D. Álvarez-Ríos, Fernando Pacheco-Torres, Carmen Julia Figueredo-Urbina, Alejandro Casas

**Affiliations:** 1 Instituto de Investigaciones en Ecosistemas y Sustentabilidad, Universidad Nacional Autónoma de México, Morelia, Michoacán México; 2 Departamento de Ingeniería Bioquímica, Tecnológico Nacional de México, Morelia, Michoacán México; 3 Cátedras CONACYT-Laboratorio de Genética, Área Académica de Biología, Instituto de Ciencias Básicas e Ingeniería, Universidad Autónoma del Estado de Hidalgo, Mineral de la Reforma, Hidalgo México

**Keywords:** Domestication, Fermented beverages, Mesoamerica, Pulque agaves, Traditional knowledge

## Abstract

**Background:**

Pulque is a fermented beverage prepared with sap of *Agave* species in Mexico. Management of agaves for this purpose has motivated domestication of some species and high phenotypic variation that commonly causes uncertainty about the taxonomic identity of varieties traditionally managed by people. This study assumed that varieties of crop species continually arise from mutations, sexual reproduction and hybridization, among other processes, and some of them are favoured and maintained by humans. Identifying these varieties may be difficult and a challenging issue for botanists and evolutionary biologists studying processes of domestication. Through a case study, we analysed the traditional varieties of agaves used to produce pulque in Michoacán, Mexico. We aimed at identifying the varieties, analysing the relatedness among them and developing a methodological approach that could help solve taxonomic problems and study variation under domestication of this and other plant groups. We documented (1) the traditional varieties of agave used and their identity, (2) how these varieties are perceived, used and managed by the local people and (3) how management influences phenotypic and genetic variation among varieties.

**Methods:**

We interviewed pulque producers in two localities of the state of Michoacán, Mexico, where we recorded management practices of agaves, the traditional varieties used, the attributes characterizing those varieties, the varieties preferred by people, and features and mechanisms of selection. We conducted multivariate analyses of morphological features of the agave varieties, as well as genetic diversity and genetic distance studies among agave varieties through 11 nuclear microsatellites.

**Results:**

Seven traditional varieties of *Agave* were recorded in the study area. Multivariate analyses of morphology identified varieties belonging to the species *A. salmiana*, *A. mapisaga* and, presumably, *A*. *americana*. The preferred varieties have morphological features selected to make easier their management and produce higher sap yields. Genetic diversities (*H*_*E*_ = 0. 470 to 0.594) were high compared with other *Agave* species with similar life history traits and use. Genetic distance analyses grouped the varieties “Verde” and “Negro” (identified as *A. salmiana*), whereas the varieties “Tarímbaro” and “Listoncillo” (identified as *A. mapisaga*) formed another group. The varieties “Blanco” and “Carrizaleño” (most probably being *A. americana*) clustered with varieties of *A. salmiana*, whereas the variety “Cenizo” appeared as a distinct group*.* Bayesian analysis indicated that most individuals of varieties of *A. salmiana* form a group and those of the varieties of *A. mapisaga* form another, whereas individuals of the varieties putatively belonging to *A. americana* clustered in similar proportions with both groups.

**Conclusions:**

The traditional pulque production in the study area is an ongoing practice. It is still an important source of products for direct consumption by households and generation of economic incomes and as part of the cultural identity of local people. The most used traditional variety exhibited a marked gigantism, and although these agaves are mainly asexually propagated, populations have high genetic diversity. The local producers promote the maintenance of different traditional varieties. Our study shows the value of an integral research approach including ethnobiological, morphological and genetic information to clarify the state of variation influenced by humans on agaves, but it would be helpful to study other organisms under domestication. In addition, such approach would help to document human and non-human mechanisms generating crop varieties managed by local people.

## Background

The genus *Agave* comprises 210 species belonging to the family Asparagaceae [1], naturally distributed from southern USA, Mexico, the Caribbean region, Central and northern South America to Colombia and Venezuela [2]. Mexico is the centre of diversification and domestication of *Agave*, with 76% of the taxa described for this genus occurring in its territory, 61% of them being endemic [3, 4]. Agaves are mainly representative of arid and semiarid ecosystems, but they also occur in mountainous temperate humid and sub-humid areas [2–5].

Because of their distribution, abundance and morphophysiological characteristics, agaves have been used by different human groups since prehistory [5–8]. The main uses of these plants, documented by archaeologists, anthropologists and ethnobotanists include fibre (leaves), food (baked leaves, stems and stalks, and boiled and cooked flower buds), beverages (fresh sap: *aguamiel*, fermented sap: *pulque* and distilled must: *mezcal*), medicine (leaves), house construction (stalks) and live fences [6–10].

Archaeological studies in caves of the Tehuacán-Cuicatlán Valley revealed that agaves were among the main components of diet of the earliest hunter-gatherer people of the region. The remains recorded suggest this fact, since these include chewed fibre of agave identified in cave floors and in human coprolites from different stratigraphic levels representing different times of human occupation of the studied caves of the region, from 12,000 to 8 000 years before the present [6–8].

Use of agaves for preparing beverages derives from the extraction of the sap, which is traditionally called *aguamiel*. This practice could have originated from the observation of nature by people, since several animals, mainly rodents and lagomorphs, drink that sap of wild agaves excavating holes at the base of their lateral leaves [11, 12]; however, clear evidence of this use are lithic tools used to favour accumulation of sap for then extraction [13–15]. The oldest record of using agave sap by Mesoamerican peoples is a scraper piece of obsidian (a concave instrument used for scraping the apical meristem for allowing the accumulation of sap) which is nearly 2 300 years of antiquity. It was found in Huapalcalco, in the current state of Hidalgo, central Mexico [12, 15]. Although this record does not allow inferring about fermentation techniques, it makes possible identifying that extraction and consumption of *aguamiel* was systematic at that time.

Pulque is the name of the beverage resulting from the fermentation of *aguamiel*, which is carried out by microorganisms naturally associated to the sap, like lactic acid bacteria and yeasts [16–19]. A simple storing of sap determines its fermentation into pulque in one or two days; therefore, the early use of *aguamiel* could have been associated to preparation of pulque. In the archaeological site of Teotihuacán, in central México, organic residuals recorded in pottery are evidence of storing and consumption of fermented sap in habitational complexes of the city, approximately between 1730 and 1450 years before the present [20, 21]. In addition, there is pictographic evidence of the extraction of sap and consumption of pulque in pre-Columbian and Colonial documents. In “La Relación de Michoacán” (Chronicles of Michoacán) [22], a document of the XVI Century, the history and traditions of the ancient habitants of southwestern Mexico are recapitulated. In that document, two paintings can be found representing the pulque agaves and the extraction of sap by the *P’urhépecha* people.

According to Colunga-GarcíaMarín et al. [23], 41 *Agave* taxa (species and intraspecific botanical categories) have been recorded to be used for extracting *aguamiel* and producing pulque in México. The information about agave management is relevant for analzing their role in past and current societies, particularly the human practices and decisions to transform, adjust, maintain or recover resources according to cultural purposes [24, 25]. Such information allows identifying that their recurrent management may lead to domestication, a process through which organisms experience phenotypic and genotypic changes, as a consequence of differential reproduction of individuals favoured by humans through conscious or unconscious selection [26]. Phenotypic attributes differentiating domesticated organisms from their wild ancestors were recognized by Darwin [27, 28], and more recently, several authors have called them domestication syndrome [29, 30]. The domestication syndrome in agaves has been influenced by human selection directed to favour features for different purposes. For instance, larger plant size favours increasing amount of edible matter and sap, while larger leaves favour increasing the amount of fibre [9]. In addition, people may favour features like decreasing the number and size of spines and removing irritating secondary metabolites, which makes easier the management of agave plants [31–33].

At least 10 species and subspecies of agaves were domesticated in Mexico, and the continuous management by traditional handlers in different regions has generated numerous traditional varieties of these plants, which have not been clearly described or classified [9, 31–36]. Traditional varieties are those variants of crops that are recognized, named, managed, propagated and preserved by the local producers and smallholders. These varieties are strongly associated with the knowledge of the producers, specific uses and purposes, but whose formal taxonomic identity is in some cases unclear. These are commonly intraspecific categories, but some of them are probably of inter-varietal or interspecific hybrid origin. Therefore, we focused our attention to characterize the agave varieties of a particular area, based on morphological, genetic and ethnobiological approaches [37–39]. In this study, we make use of the term traditional varieties to distinguish them from varieties and cultivar categories defined by the formal plant taxonomy.

In species and traditional varieties of agaves used for sap consumption, the features favoured by human selection are (1) larger amount and quality of sap (higher content of sugars), (2) gigantism of the plant size, which is related with production of a higher amount of sap, (3) decreasing number and size of spines and leave teeth, (4) low concentrations of saponins and (5) high production of vegetative propagules to make easier and more intensive cultivation of the desirable phenotypes [9, 31–33, 40, 41].

A study that illustrates how complex the traditional varieties of agave may be was that by Mora-López et al. [36], who evaluated the morphometry of species of the Section Salmianae, considering varieties of *Agave macroculmis* Tod, *A. mapisaga* Trel. and *A. salmiana* Otto ex Salm, in the Altiplano Meridional (Southern Highlands) of the Central-Northern region of México. These authors recorded 62 traditional varieties of agave, with *A. mapisaga* having plants with the largest size and lowest number and size of teeth, while *A*. *macroculmis* had the lowest size and greater teeth number and size, and *A. salmiana* had the highest morphological variation.

The state of Michoacán is the setting of the traditional preparation of pulque by the Puhrépecha and offers an interesting cultural area for studying variation of agaves associated to ancient management. The species most widely used for producing pulque are *A. salmiana*, *A. mapisaga, A. americana* L. and *A. hookeri* Jacobi. The latter species is endemic of the region, but it is rarely used at present; it can be found at low densities in live fences and its use is considered almost lost [5, 9]. Recent studies identified that *A. hookeri* shows clear signs of domestication, notably gigantism, and loss of genetic variation compared with its putative wild ancestor *A. inaequidens* Koch [31, 32].

The intensive management of agaves for producing pulque and other purposes has motivated human selection and domestication determining high morphophysiological variation that frequently causes uncertainty about the taxonomic identity of the traditional varieties of agave. Identifying traditional varieties is part of the process of understanding how those varieties are associated to human management. In agaves, as in all crop species, domestication continually generates new varieties, but sometimes it is difficult to know their nature and their relation to cultural values [42]. Therefore, identifying affinities of traditional varieties to some species may allow explaining their origin and relations, which is in turn important for characterizing and managing genetic resources.

Genetic diversity of agave populations is generally high, whereas genetic differentiation is low [43]. But patterns vary depending on their natural history, as well as their type of use, management and domestication. One of the extreme cases is the almost null genetic variation in the intensive managed *Agave tequilana* Weber, which contrasts with the high levels of genetic variation exhibited by populations or their wild relatives in forests and those maintained in agroforestry systems [34, 44]. For some agaves, used for producing pulque, studies have reported low genetic diversity, high structure and low gene flow [32, 45], and all these patterns are possibly responses to selection favouring certain phenotypes, vegetative propagation and limited sexual reproduction [32]. Biological populations differentiate according to the types and frequencies of alleles and genotypes. Such differentiation is called genetic structure. It is particularly high when gen flow among populations is low. Parameter *F*_*ST*_ evaluates structure in a range of values between 0 and 1, the minimum and maximum values of differentiation or structure, respectively. The populations studied differentiating between 0 and 0.05 represent very low structure, those between 0.05 and 0.15 express moderate structure, and those between 0.15 and 0.25 have high, whereas those > 0.25 have very high structure [46, 47].

The ancient and current processes of domestication of agaves have determined progressive diversification, which in addition to hybridisation and local adaptation have contributed to generate complexes of phenotypic variation, whose taxonomic identity is commonly difficult to characterize. According to the use of agaves, the domestication syndrome may have different expressions, which may leave prints of past or current processes of human selection and may be helpful for characterizing the potential of genetic resources. Such situation makes necessary to carry out efforts for identifying specific and intraspecific taxa, documenting the state of variation and the evolutionary relations among taxa, in order to understand the origin of such variation, the natural and human factors influencing it and the optimum conditions for designing strategies of conservation of genetic resources considering all such factors.

The aims of this study are to (1) document ethnobotanical aspects on use of traditional varieties of agaves and preparation of pulque in localities of Michoacán, central Mexico, (2) document the perception of the agave varieties by local people, their particular attributes, management types and mechanisms of selection and maintenance and (3) characterize morphological and genetic similarities of the agave varieties, and a more precise characterization of the domestication syndrome of these plants. We hypothesized that phenotypical features resulting from human selection are reflected in the traditional varieties recognized by local people based on their attributes, forms of use and management types. In addition, the varieties more intensely managed would exhibit the clearest features of domestication syndrome.

## Materials and methods

### Species studied

*Agave salmiana* belongs to the section Salmianae, which is widely cultivated in the states of Tlaxcala, Michoacán, Aguascalientes, San Luis Potosí, Hidalgo, Oaxaca and Puebla. In the latter states, it is possible to find small wild individuals that are likely ancestors of the crops. *Agave mapisaga* is larger than *A. salmiana*, and it is common to find them growing together. It is mainly found in the states of México, Morelos, Puebla, Michoacán and Zacatecas, but in addition, we have registered it in the states of Hidalgo and Tlaxcala. *Agave americana* is found in the states of Nuevo León, Michoacán and Oaxaca. Because these species are cultivated together, hybridization among the cultivars is likely. The propagation of these species is mainly vegetative through suckering rhizomes or by sprouts in the corm. Sexual reproduction is limited or null, since people cut the apical meristem to harvest the sap, thus preventing the development of the inflorescence [5]. These three species are widely cultivated [5, 10].

### Study area

**Fig. 1:**
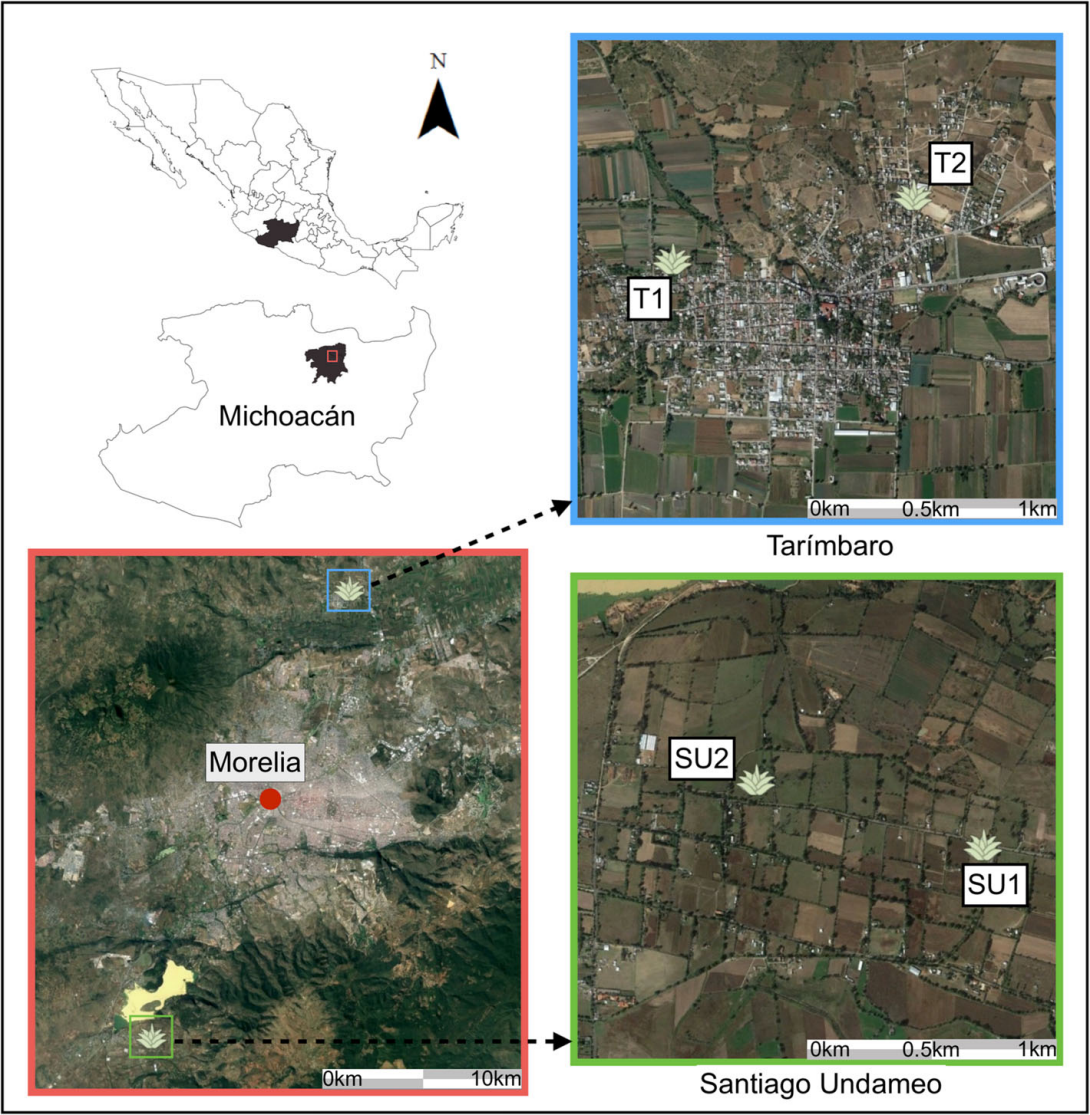
Map of the studied localities, Tarímbaro (T) at the north and Santiago Undameo (SU) at the southwest of the city of Morelia. Units of cultivation of agave where the samples for genetic analysis were collected and morphological measurements of agaves were conducted are indicated as T1–T2 in Tarímbaro and SU1–SU2 in Santiago Undameo

Our study was conducted in the state of Michoacán, where people have used agaves and prepared pulque since pre-Hispanic times, as revealed by several pictographic sources of the XVI Century [12, 22]. However, few studies have documented past and current production systems in this area. We studied in two localities that use and manage agaves for preparing pulque; one is the locality of Tarímbaro (T) a pre-Columbian town, with historical records for the local people’s life, the ancient production of pulque and the management of agaves [48]. T is located at the north of the city of Morelia, in the Municipality of Tarímbaro (Fig. 1), where annual mean temperature is 22 °C and annual precipitation 600–800 mm, the rainy season occurs from May to October [49]. The locality is part of the Metropolitan Area of Morelia, and although it has a long tradition of pulque production, it has dramatically decreased because of the urban expansion and reduction of spaces for agave cultivation [48].

The other locality is Santiago Undameo (SU), located in the municipality of Morelia at the southwest of the city (Fig. 1), where annual temperature is on average 17 °C, and precipitation 800–1000 mm per year, with a rainy season from May to October [49]. Human activities are predominantly agriculture, farming fields and pastures for livestock, and cultivation of agave for preparing pulque.

Both communities studied are the main providers of pulque to the city of Morelia. It is particularly important to study management of agaves and pulque in this region because it has been insufficiently studied compared to the production systems of pulque in other parts of central Mexico. We centered our attention on local varieties of some *Agave* species or interspecific hybrids to develop a methodological approach helpful for studying other species of *Agave* and crop plants.

### Ethnobotanical assessment

Semi-structured interviews were conducted with most of the *pulque* producers in the villages studied (seven in T and five in SU), which allowed documenting management systems of pulque agaves, description of the traditional agave varieties, characterizing their distinctive features, and the quality and quantity of their sap, as well as the management of the sap to prepare pulque.

### Morphometric studies

We measured 72 agaves of different traditional varieties. These plants were selected according to their development stage, all of them mature agaves ready to extract their sap, identified according to the criteria of the local producers. Then, 12 measurements were conducted in vegetative parts following Figueredo et al. [31], and eight relations among some variables were estimated. In total, 20 morphological characters were analyzed. The closest taxonomic identity was approached based on the taxonomic keys developed by Gentry [5].

### Statistical analysis

Tests of normality and variance equality were performed for all datasets, and based on such tests, we decided to perform non-parametric analyses (Kruskal Wallis test) and paired comparison through IBM-SPSS Statistics 22, in order to identify how morphological characters studied differed between the varieties studied. Principal component (PCA) and cluster (CA) analyses were carried out, with the purpose of classifying the sampled populations and individual plants. We used the software R (v. 3.5.0), and because of differences associated to the type of character and the measurement units of the variables, we standardized the data matrix using the function *scale* (mean-centering). In addition, Bootstrapping was carried out to evaluate the optimum number of clusters and the cohesion between them, represented by the Jaccard index (0 represents a null cohesion and 1 a high cohesion).

### Genetic diversity

Samples of agave tissue were collected from healthy leaves, dried and stored on silica gel until DNA extraction. Total DNA was extracted using CTAB extraction protocol for plants [35]. Eleven nuclear microsatellite loci ([50, 51], Cabrera-Toledo et al., unpublished data) were used in three mix Multiplex (Table 1), considering the fluorescent marker and the size of the amplification obtained by Pacheco-Torres [52]. Polymerase chain reactions (PCRs) were performed according to the indications for Mix Platinum Polymerase (Thermo-Fisher). Amplifications were carried out in a MultiGen thermocycler (LabNet) under the following conditions: heat activation of 15 min at 95 °C, 35 cycles: denaturation at 95 °C for one min, alignment at 60 °C for one min, and extension at 72 °C for 90 s. A final extension step at 72 °C for 7 min was included. We took 1 μL of product from each PCR and mixed it with 10 μL highly deionized formamide (Hi-Di Formamide, Applied Biosystem) and 0.3 μL of standard size marker Gene Scan LIZ-500 (Applied Biosystem); prior to the analysis, a denaturation was carried out at 95 °C for 5 min and then analyzed by capillary electrophoresis in a Genetic Analyzer 3130xl sequencer (Applied Biosystems). Electropherograms were analyzed using the Peak Scanner™ Software v1.0 program (Applied Biosystems). Using GenAlEx [53], we estimated the number of effective alleles (*A*_E_), observed heterozygosity (*H*_O_) and expected heterozygosity (*H*_E_). We made a dendrogram of the MEGA program [54], according to the UPGMA method, using genetic distances paired of Nei [55] between the varieties. We also performed a Bayesian grouping with STRUCTURE [56]. The optimal number of genetic groups was determined by varying the *K* from 1 to 8 with 10 runs for each *K.* Every run consisted of 10,000 burn-in and 50,000 periods of MCMC repetitions after the burn-in. The number of subpopulations was additionally estimated based on the approach of Evanno et al. [56] using Structure Harvester [57]. In order to align the cluster membership coefficients of the ten Structure runs and to graphically display the results, we used CLUMPP version1.1.2 [58] and Distruct version1.1 [59].

**Table 1:** Characteristics of the 11 nuclear microsatellites used in genetics analysis for species domesticated agave in the state of Michoacán, Mexico

Locus	Flourescents dye	Allele size range (bp)	Multiplex set	Reference
P1-5G	NED	151-169	1	Parker et al. 2014 [35]
APAR2-12	VIC	155-164	1	Lindsay et al. 2012 [51]
APAR3-11	6-FAM	126-218	1	
APARLC20	PET	134-158	1	
APARLC21	NED	246-252	1	
APARLC28	NED	151-193	2	
APARLC34	PET	168-174	2	
APARLC35	6-FAM	170	2	
P1763	6-FAM	176-222	3	Cabrera-Toledo et al. unpublished data
P1448	VIC	122-158	3	
P1659	PET	158-178	3	

## Results

### Ethnobotany of agave management

**Fig. 2:**
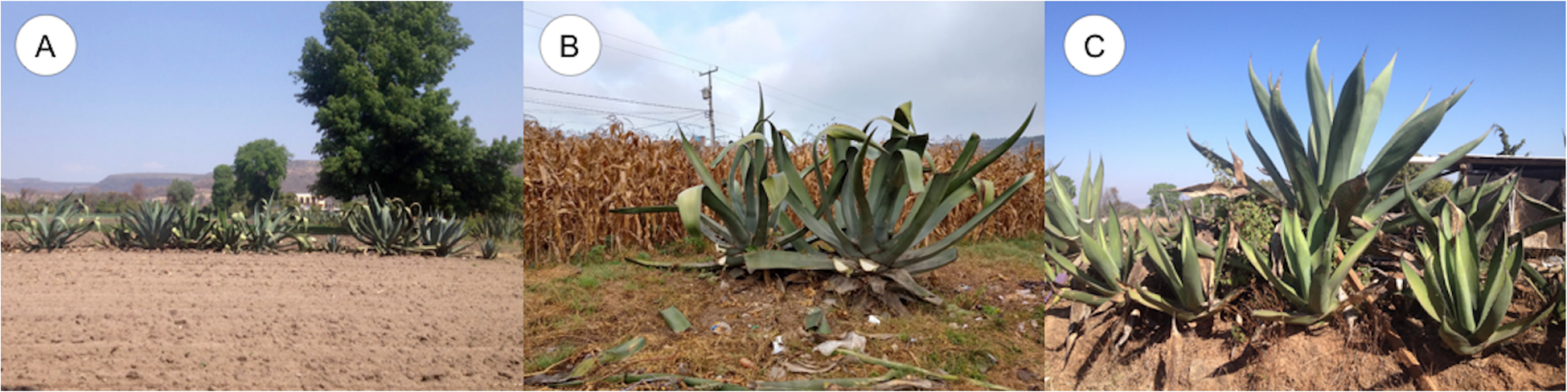
Traditional varieties of agave used for producing pulque in the state of Michoacán, **a** Agaves delimiting a field without cultivation (varieties “Listoncillo,” “Blanco” and “Verde”). **b** Agaves (“Listoncillo”) at the edge of a cornfield. **c** Agaves (“Verde”) at the edge of a road

The units of agave cultivation studied are located within agricultural matrixes (areas with crops, without crops, pasture areas, houses and roads). The matrix of each producer is 2.5–4 ha extent. The agaves function as living fences: (1) delimiting parcels surrounding the crop fields, which is the most frequent condition; (2) in spaces where the land is not suitable for agriculture, small patches of vegetation where agaves are grown; (3) roads and paths bordered with agaves (Fig. 2a–c). The main activities practiced by the pulque producers are agriculture (maize, beans, squashes and alfalfa), raising of animals (cows, sheep and/or chickens) and commercializing the *aguamiel* and pulque they produce, both within the community and bringing their product to sell in the city of Morelia. The main purpose people mentioned to grow agaves is to obtain *aguamiel* and pulque; in addition, the capacity of these plants to retain moisture and soil, their effectiveness to define limits of their croplands and preventing entering people or animals that may cause damage in their land and crops.

**Fig. 3:**
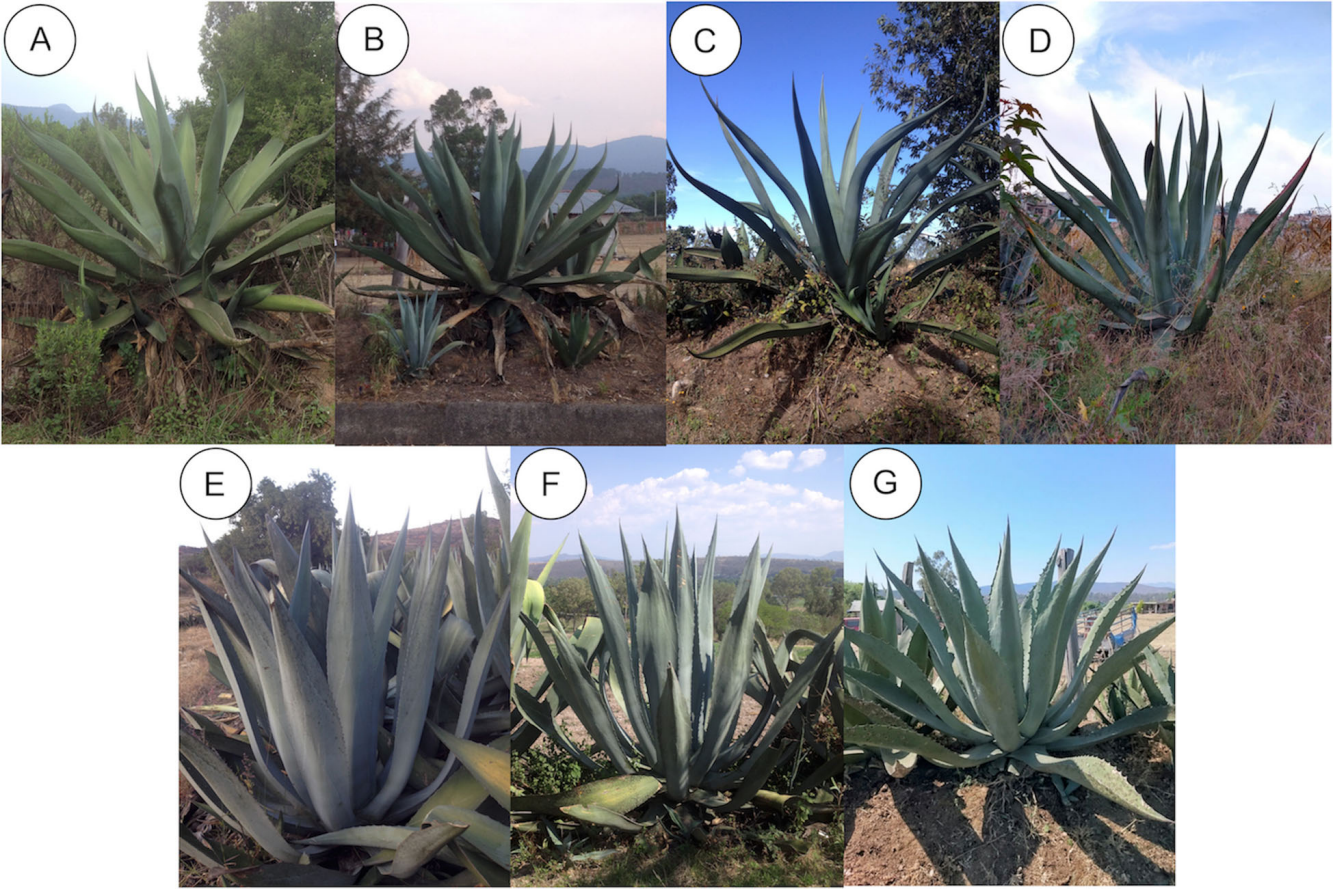
Traditional varieties of pulque agaves from the localities of Santiago Undameo and Tarímbaro, Michoacán. **a** Verde: *Agave salmiana* var*. salmiana*; **b** Negro: *A. salmiana* var*. salmiana*; **c** Tarímbaro: *A. mapisaga* var. *mapisaga*; **d** Listoncillo: *A. mapisaga* var. *mapisaga*; **e** Blanco: *A.* aff. *americana*; **f** Carrizaleño: Most probably *A. americana* subsp. *americana*; **g** Cenizo: Most probably *A. americana* var. *americana*

**Fig. 4:**
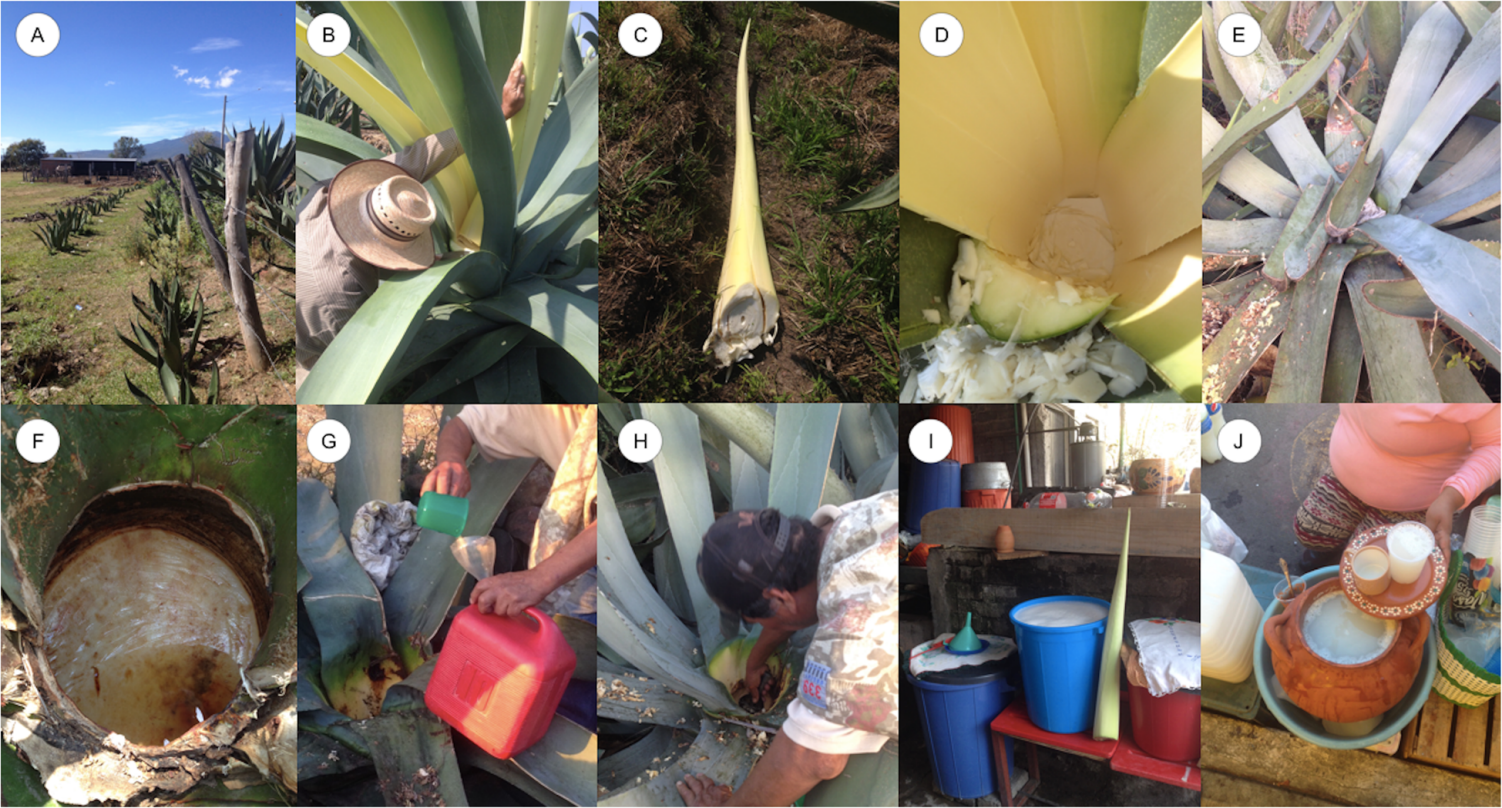
Management practices in Tarímbaro and Santiago Undameo, Michoacán. **a** Cultivation of agaves put in line, one year after transplantation. **b** Start of sap extraction process, the producer cuts the “heart” (central stem) of a mature agave. **c** The cut stem (*maguey’s heart*). **d** Cavity excavated (*cajete*) in the centre of the agave, where sap will accumulate. **e** Cavity covered with leaves of the same agave. **f** Cavity uncovered with accumulated sap. **g** Sap collection. **h** Scraping of the agave cavity in order to make the sap flows. **i** Sap fermentation in plastic barrels. **j** Pulque sale outside the producer’s home

The general common name for all agaves in the localities studied is “maguey.” All producers have their own agaves; however, it is common that the standing plants ready to produce sap are sold (more precisely, the right to extract the sap from them) among producers. The price of a *maguey* ready to produce pulque may be $350 pesos (nearly 18 U.S. Dollars) if the plant measures 2 m in height, $500 (nearly 25 U.S. Dollars) if it is between 2.5 m and 3 m and $700 (nearly 36 U.S. Dollars) if it exceeds 3 m in height. The producers identify and manage different traditional varieties of agaves for pulque: “Verde,” “Negro,” “Tarímbaro,” and “Cenizo” in SU; “Verde,” “Listoncillo,” “Blanco” and “Carrizaleño” in T (Table 2, Fig. 3). These traditional varieties are named based on their morphology, mainly their general plant size, leaves colour and arrangement, thorns and lateral teeth, as well as quantity, sweetness and period of sap production. The practices of propagation, cutting apical meristems and sap extraction are similar among the households interviewed in the two communities. For using these agaves, the producers propagate them by transplanting the suckers, which grow around the mother plant and when they are 30 cm in height and the producers interviewed judge that they are vigorous enough because “they look resistant and strong;” they thus transplant them to another site where they can grow without competition within the spaces previously described for cultivation (Fig. 4a).

**Table 2:** Characteristics of the traditional varieties of agaves identified by producers in the state of Michoacán

Traditional variety name	Main features	Location (measured plants)	Taxonomic identity
*Verde*	It is the most widely used variety, for the quantity and quality of its sap, produces on average 3 L a day, for 4 months and is valued for the high sweetness of its sap. This maguey exceeds 2 m in height, is leafy, with broad leaves and mostly grooved towards the tip, and has lateral spaced teeth and a prominent terminal thorn. Its coloration is an intense green and is very abundant in both locations.	SU and T (*n* = 17)	*Agave salmiana* var. *salmiana* Álvarez-Ríos collection 7, 8
*Negro*	It is a variety recognized by the attributes that are similar to “Verde” variety; it is only distinguished because it has a darker green coloration. Just one producer identifies it in SU, the rest do not distinguish between “Verde” and “Negro” variety.	SU (n = 18)	*A. salmiana* var*. salmiana* Álvarez-Ríos collection 9, 10
*Tarímbaro*	This variety is little used in SU, so its abundance is reduced, producers say that the sap is not as sweet as Verde variety, but produces more, about four liters on average per day. It is larger and reaches 3 m high; its leaves are thin and long, so they lose rigidity. The lateral teeth are small and seem to disappear. It receives the name of Tarímbaro since according to the producers this variety was brought from that locality.	SU (n = 2)	*A. mapisaga* var. *mapisaga* Álvarez-Ríos collection 13, 14
*Cenizo*	This variety is not abundant in SU, only two individuals were registered. It is little used since it is affirmed that the sap is insipid and the produced pulque is not so good. This variety has a grayish appearance, hence the name "Cenizo." It is smaller than other varieties, and it does not reach 2 m high, with small erect leaves; it has big lateral teeth with a hook shape. It produces one and a half liters per day.	SU (n = 2)	Most probably *A. americana* var. *americana* Álvarez-Ríos collection 1, 2
*Listoncillo*	Its characteristics are the same as “Tarímbaro” variety. People name it “Listoncillo,” because of its length, it is thin, and its fallen leaves, which resemble a ribbon. Producers also claim that their sap is less sweet than “Verde” variety, it gives 4 L on average per day for five months. In T, it is as abundant as “Verde” variety; it is common to find exclusive accumulations of this variety.	T (n = 27)	*A. mapisaga* var. *mapisaga* Álvarez-Ríos collection 11, 12
*Blanco*	It is a grayish variety, with slightly grooved and erect leaves. It exceeds two meters in height, its lateral teeth are hooked and has a big terminal spine. It is less used than “*Verde”* and “*Listoncillo”* variety because the aguamiel is not as rich (tasty), since for producers it is less sweet. It produces one and a half liters per day.	T (n = 2)	*Most probably A. americana* var. *americana* Álvarez-Ríos collection 5, 6
*Carrizaleño*	It is a grayish agave, with slightly flat and erect leaves that have a peculiar widening in the middle part. It is about 2 m high, without being leafy. They are particularly fibrous, as a person interviewed said "they are very hard, they are like reeds," hence their name. It has hooked lateral teeth. Its sap is very sweet at the beginning of production, but after a month and a half or 2 months, according to people interviewed, it becomes insipid "it becomes like water". This is the variety that is in lowest proportion in T. It produces one and a half liters per day.	T (n = 4)	*Most probably A. americana* var. *americana* Álvarez-Ríos collection 3, 4

*SU* Santiago Undameo, *T* Tarímbaro, *according to Gentry 1982

The pulque producers never collect the seeds of agave to propagate them, and it is rare to let the agaves bloom, as people interviewed said “... only [they leave standing] some agaves forgotten...” since using the agaves the inflorescences have to be removed before their development. According to people, only agaves occurring in areas of difficult access, or those that are unnoticed, or those deliberately left to develop flowers and fruits may bloom. The pulque producers affirm that an agave is ready to produce pulque when it is 8 to 10 years old. This can be distinguished by indicators such as the thinning of the apical meristem, “the maguey heart (central apex) becomes very thin;” the leaves furthest from the meristem become expanded while those closer enclose the meristem. The pulque producers interviewed describe this stage saying that “the maguey starts to be like a sponge.” Other producers identify this stage based on the number and size of leaves. For instance, one person interviewed said: “you can see when it is good to produce because it has exceptionally large leaves, it is well leafless and then you count the leaves, if they are 40 it is already good.”

Sap extraction is carried out when an agave is mature, and at that stage, the apical meristem is cut off, generating a cavity where the sap flows and accumulates. The producers firstly remove a couple of leaves to make easier the access to the centre of the plant, then they cut the edges of the adjacent leaves to avoid scratches with the lateral teeth. Following this procedure, the meristem is cut from the union with the first leaves, then excavated to form the cavity called “cajete” (Fig. 4b–d). When the *cajete* is ready, it is covered with the cut leaves and stones; the collection of sap starts during the days after this activity (Fig. 4e).

The *aguamiel* is collected with a plastic cup and then poured in a bucket. After each event of sap collection, it is necessary to scrape the *cajete* with a tool called “raspador,” which is a sharp metal spoon used to remove the tissue that forms as a scar (Fig. 4f–h). Producing this injury to the plant allows the sap flowing to be collected. In the first days of this activity, the sap is scarce, people interviewed said that “it produces just a small glass,” which is not consumed because people say it is bitter, but after 3 days the sap increases its volume and quality. An agave may produce 2.5 to 4 L per day, for 3 months on average, and up to 5 months the larger plants. The agave produces *aguamiel* until it dries; this is when all the leaves lose their turgidity and wither and the sap stops flowing.

Some producers carry out agaves scraping and collecting of their sap once or twice per day during the dry season (March to May); they consider it is better to extract twice per day because heat accelerates the process of fermentation of the sap and it may become sour when stored, but during the cold season (from November to February) they do not have this problem. Producers who collect agave sap once per day carry out this activity in the morning, from 05:00 to 07:00, while people who collect twice per day carry out the second sap collecting in the afternoon, from 17:00 to 19:00.

According to producers interviewed, the best time for pulque production is the dry season (from October to April), because during the rainy season (May to September) water may infiltrate the agave tissue and that condition decreases the quality of the sap to be fermented and the quality of pulque flavour. During the rainy season, the producers tie a plastic cover over the agaves to prevent that water introduces into the cavity where the sap accumulates.

Each producer has 10 to 15 agaves producing at the same time, from these agaves in total they extract on average 32 ± 2.7 L in T (*n* = 7) and 27 ± 2.2 L in SU (*n* = 5) per day during the dry season. While in the rainy season in SU people only extract 10 to 15 L per day, until the plants stop producing *aguamiel*. In T, the producers interviewed said to extract about 10 L less than in the dry season, but all of them continued the activity in both seasons.

After sap is collected, it is transported to the producers’ houses, into spaces specially destined to the elaboration of pulque. The elaboration of pulque in T occurs in fresh rooms, with little exposure to sunlight, and people consider it convenient to maintain low temperatures for a good preservation of the beverage. People maintain the “pie de pulque” (“foot of pulque”), which is the remaining sediment from pulque prepared the day before, generally less than 1 L, which is used for enhancing. A woman interviewed said that she adds foot of pulque since with it “the aguamiel works faster and makes pulque tastier.” According to the producers, after 3 h of fermentation, the pulque is ready to be consumed (Fig. 4i).

In SU after collected, *aguamiel* is strained and placed in a pot over a fire, to give it a slight boil (no more than one minute). As soon as the *aguamiel* begins boiling, people remove it from the heat and let it cool for an hour, then mix it with the *foot of pulque*. The drink is fermented for a couple of hours, the effervescence takes place and after 2 h it is considered that the pulque is ready to be consumed. According to pulque producers of SU, the sap generates an irritation called “carame,” then by boiling the *aguamiel* they avoid that irritation.

Once the pulque is ready to be consumed, the producers sell it in their homes or in the *tianguis* (itinerant markets) or established markets. The average price is $20 pesos (nearly 1 U.S. dollar) per L in SU and $16 pesos for T (nearly 0.8 U. S. dollars) per L (Fig. 4j).

### Morphological variation of agaves

The interviews allowed identifying the following traditional varieties of agave: “Verde,” “Negro,” “Tarímbaro,” and “Cenizo” in SU and “Verde,” “Listoncillo,” “Blanco,” and “Carrizaleño” in T (Table 2, Fig. 3). The most used variety of agave in SU is the “Verde,” while in T the most used are the “Verde” and the “Listoncillo.” The varieties “Cenizo” and “Tarímbaro” in SU, and the “Blanco” and “Carrizaleño” in T, are used in smaller proportion, because the sap of these varieties is less valued than that of the other varieties, also because plants of these varieties are perceived to have higher dentition. However, people said they use these varieties when the preferred ones are scarce.

Table 2 summarizes data for the characters measured in the seven traditional varieties identified. For all the characters, we found significant differences between the varieties (*p* ≤ 0.001). The main trends were a greater general size of plants (GLP), greater average diameter (D) and leaf length (LL) of the “Tarímbaro” and “Listoncillo” varieties. Differences between these varieties for the 20 characteristics analyzed were not significant, suggesting that they both correspond to the same species, according to the general morphology, *A. mapisaga.* var. *mapisaga* (the rosettes are nearly 3 m in height, and the diameter of up to 4 m, the leaves are linear with a length greater than 2 m). These varieties are followed in size by the “Verde” and “Negro” varieties, which, according to the comparisons, were not significantly different among themselves, and whose general morphology corresponds to *A. salmiana* var*. salmiana* (the leaves are lanceolate, the rosette small, with large teeth). A noticeable difference of these varieties with those of *A. mapisaga* var. *mapisaga* is that leaves are significantly wider (LW).

We found that the varieties “Cenizo,” “Blanco” and “Carrizaleño” have characters like leaf width (LW), leaf length (LL) and general plant length (GPL) resembling *A. salmiana* var. *salmiana*, but other characters such as the shape of leaves, dentition, terminal thorn length (TTL) and plant colour are similar to *A. americana* var. *americana*

Regarding characters associated with dentition, even when *A. mapisaga* var. *mapisaga* exhibited more teeth, which is associated with the larger size of its leaves, these teeth and the terminal spine were smaller than those of the other varieties. The terminal spine of *A. salmiana* var. *salmiana* was longer, with more and larger teeth than all agaves here studied, while *A. americana* has large teeth, but more spaced among them along the margin of the leaf, exhibiting strong differences in these characters with *A. mapisaga* var. *mapisaga*.

**Fig. 5:**
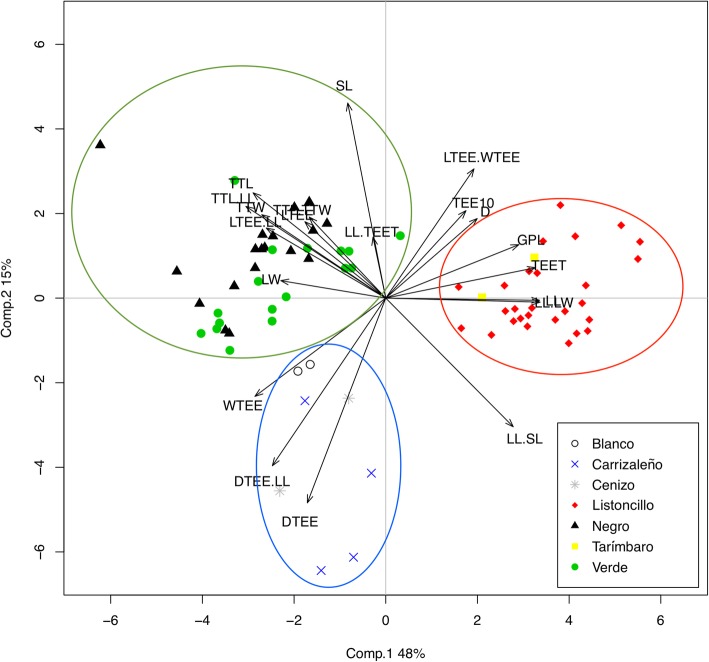
Principal components analysis (PCA) according to the 20 morphological variables measured in the seven traditional varieties of *Agave* in the studied localities in the state of Michoacán. The varieties “Tarímbaro” and “Listoncillo” form the red circle group. The varieties “Verde” and “Negro” are enclosed in the green circle group and “Carrizaleño,” “Cenizo” and “Blanco” in the blue circle group

In the PCA performed, three groups of agaves were identified based on their morphological characters (Fig. 5), the first component explains 48% of the variation, while the second explains 15%. The ordination pattern shows a first group in the sector on the right (inside the red circle), composed by individuals of the varieties “Tarímbaro” and “Listoncillo,” classified by the most numerous but smallest teeth (TEET), the length of the leaf (LL), the total height of the plant (GPL) and the average diameter (D), all characters defining the largest sized plants. The second group, comprised in the green circle of the plot (Fig. 5), is composed by individuals of the varieties “Verde” and “Negro,” which are plants smaller than those of the red circle group, with leaves shorter but wider (LL/LW), the length of the stem is greater and they have a larger terminal spine. The varieties “Carrizaleño,” “Cenizo” and “Blanco” are in the lower left part of the plot (blue circle) (Fig. 5); plants of this group have smaller size, with a more pronounced dentition than the other groups; such dentition characters are associated to the width of the lateral teeth (WTEE) and the distance between them (DTEE). The analysis of Bootstrapping showed three groups. The group inside the red circle has a Jaccard coefficient of 0.75, which confirms that it is the most cohesive and homogeneous group. For the groups inside the green and blue circles, the coefficients are 0.61 and 0.12, respectively, the latter indicating a very low cohesion coefficient.

Based on the analyses carried out and the review of taxonomic descriptions [5], we can say that the varieties “Listoncillo” and “Tarímbaro” may belong to *Agave mapisaga* var. *mapisaga*. It is the species of the genus with the largest sized individuals, characterized for its elongated and generally folded leaves, as well as small lateral teeth ranging from 2 to 5 mm in length [5]. The varieties “Verde” and “Negro” may be *Agave salmiana* var. *salmiana*; although the varieties are identified as different due to their colour, the morphometry of the plants is similar to *A. salmiana* var. *salmiana*, which is characterized by its large size, although smaller than *A. mapisaga*, a spaced dentition and the peculiar shape of its leaves: broad, convex at the base and concave upward with the sigmoid curved apex [5].

Regarding the rest of the varieties used in the area, the taxonomic identification does not correspond clearly with features of *Agave americana*, which according to Gentry [5] has lanceolate leaves, narrowed towards the base, rounded down, which from the upper 2/3 of the leaves these tend to flatten and are firm, thick, straight and ascending; it has lateral teeth in a remarkable hook and terminal thorn of 3 to 6 cm in length, in addition to its glaucous tones. With the features listed above and the analyses carried out, we propose that the varieties “Blanco,” “Carrizaleño” and “Cenizo” correspond to *Agave americana* but not clearly defined.

### Genetic variation

The locus APARLC35 was monomorphic for all species and varieties in both localities. We found heterozygous fixation at the loci P1-5G and APARLC34 of the varieties “Negro,” “Verde” (*Agave salmiana* var. *salmiana*), “Tarímbaro” and “Listoncillo” (*A. mapisaga* var. *mapisaga*). The number of effective alleles (*A*_E_) ranged from 1.000 to 4.728. The individuals of *A. salmiana* var. *salmiana* exhibited an average value of *A*_E_ of 2.334 ± 0.360. In the case of *A. mapisaga* var. *mapisaga*, the average value of *A*_E_ was of 2.794 ± 0.285 and for plants putatively of *A. americana* was 2.734 ± 0.453. At the level of the varieties of agaves, the values ranged from 1.661 for “Blanco” to 2.739 for “Listoncillo” (Table 3).

**Table 3:** Vegetative morphological characteristics and genetic diversity of the seven traditional varieties of agaves, identified by producers in the localities of Santiago Undameo and Tarímbaro, Michoacán

Vegetative character	*A. salmiana* var*. salmiana*		*A. mapisaga* var. *mapisaga*		Most probably *A. americana* var. *americana*			PC1	PC2
	*Verde*	*Negro*	*Tarímbaro*	*Listoncillo*	*Blanco*	*Cenizo*	*Carrizaleño*		
General plant lenght (GPL)	209.18 ± 4.12a	214.17 ± 5.87a	269.00 ± 11.00ab	274.68 ± 4.31b	238.00 ± 12.00ab	167.00 ± 13.00a	218.00 ± 10.17ab	0.264	0.115
Stem lenght (SL)	74.06 ± 3.63a	80.44 ± 3.67ab	67.50 ± 2.50abc	66.00 ± 2.36bc	55.00 ± 7.00abc	50.50 ± 5.50abc	46.25 ± 3.12c	− 0.075	0.419
Mean diameter of the plant (D)	296.82 ± 12.31ac	294.06 ± 8.28ac	411.75 ± 42.25b	363.57 ± 13.88b	392.00 ± 9.50b	266.25 ± 3.75 ac	257.63 ± 18.90c	0.181	0.171
Leaf lenght (LL)	153.21 ± 3.66a	137.06 ± 5.79a	203.00 ± 19.00ab	229.32 ± 3.74b	168.50 ± 2.50ab	125.50 ± 9.50a	167.75 ± 6.28ab	0.305	− 0.005
Leaf width at middle (LW)	26.50 ± 0.40a	27.17 ± 0.63a	24.00 ± 0.00ab	22.69 ± 0.46b	29.50 ± 0.50ab	21.50 ± 0.50ab	27.75 ± 2.29ab	− 0.208	0.038
LL/LW	5.79 ± 0.12a	5.10 ± 0.24a	8.46 ± 0.79ab	10.26 ± 0.30b	5.71 ± 0.01ab	5.85 ± 0.58ab	6.18 ± 0.58ab	0.306	− 0.009
LL/SL	2.12 ± 0.07a	1.75 ± 0.10abc	3.02 ± 0.39abcd	3.57 ± 0.10c	3.11 ± 0.35abcd	2.49 ± 0.08abcd	3.68 ± 0.31d	0.253	− 0.276
Terminal thorn length (TTL)	7.30 ± 0.24a	6.87 ± 0.18a	3.88 ± 0.12abc	3.29 ± 0.07b	4.13 ± 0.62abc	3.34 ± 0.34abc	3.26 ± 0.11c	− 0.263	0.226
Terminal thorn width at the base (TTW)	1.09 ± 0.03a	1.17 ± 0.04ad	0.57 ± 0.01b	0.70 ± 0.02cb	1.13 ± 0.12abcd	0.65 ± 0.02abcd	0.68 ± 0.03bcd	− 0.246	0.179
TTL/TTW	6.75 ± 0.28a	6.00 ± 0.28a	6.81 ± 0.03ab	4.78 ± 0.14b	3.76 ± 0.96ab	5.18 ± 0.65ab	4.85 ± 0.25ab	− 0.152	0.174
TTL/LL	0.05 ± 0.00 a	0.05 ± 0.00 a	0.02 ± 0.00 ab	0.01 ± 0.00 b	0.03 ± 0.00 ab	0.03 ± 0.00 ab	0.02 ± 0.00 ab	- 0.277	0.197
Total number of teeth (TEET)	38.94 ± 3.39 a	33.50 ± 1.07 a	65.50 ± 1.50 ab	79.19 ± 2.56 b	37.00 ± 1.00 ab	36.50 ± 3.50 ab	35.75 ± 1.80 a	0.296	0.065
TEET/LL	0.26 ± 0.02 ac	0.27 ± 0.01 ac	0.33 ± 0.02 ab	0.35 ± 0.01 b	0.22 ± 0.00 abc	0.29 ± 0.01 abc	0.21 ± 0.01 c	- 0.025	0.131
Number of teeth in 10 cm^2^ (TEE10)	3.00 ± 0.17 ac	3.11 ± 0.11 ac	4.00 ± 1.00 ab	4.03 ± 0.26 ab	2.00 ± 0.00 c	3.00 ± 0.00 abcd	2.50 ± 0.29 cd	0.159	0.188
Teeth length (LTEE)	1.45 ± 0.06a	1.82 ± 0.07abc	1.06 ± 0.28abc	1.32 ± 0.07bc	2.39 ± 0.06abc	1.19 ± 0.05abc	1.00 ± 0.25c	− 0.160	0.164
LTEE/LL	0.01 ± 0.0001ab	0.014 ± 0.002bd	0.005 ± 0.001abcd	0.006 ± 0.00c	0.014 ± 0.00abcd	0.01 ± 0.002abcd	0.006 ± 0.002d	− 0.237	0.150
Teeth width (WTEE)	0.58 ± 0.04a	0.57 ± 0.02a	0.31 ± 0.07ab	0.32 ± 0.02 b	0.80 ± 0.08a	0.60 ± 0.11ab	0.70 ± 0.08a	− 0.259	− 0.211
LTEE/WTEE	2.64 ± 0.19abc	3.25 ± 0.11 ac	3.38 ± 0.16abc	4.20 ± 0.18 b	3.00 ± 0.23abc	2.05 ± 0.30abc	1.54 ± 0.51abc	0.175	0.278
Distance between teeth (DTEE)	2.05 ± 0.14a	1.78 ± 0.11a	1.16 ± 0.29ab	1.15 ± 0.09b	3.02 ± 0.25ab	3.33 ± 0.61ab	4.41 ± 0.65a	− 0.156	− 0.440
DTEE/LL	0.01 ± 0.00a	0.01 ± 0.00a	0.01 ± 0.00ab	0.01 ± 0.00b	0.02 ± 0.00ab	0.03 ± 0.01a	0.03 ± 0.00a	− 0.225	− 0.360
Effective number of alleles (A_E_)	2.230 ± 0.273	2.183 ± 0.313	2.079 ± 0.166	2.739 ± 0.261	1.661 ± 0.321	1.667 ± 0.389	2.316 ± 0.397		
Observed heterozygosity (H_O_)	0.602 ± 0.120	0.586 ± 0.118	0.682 ± 0.102	0.645 ± 0.108	0.364 ± 0.136	0.364 ± 0.136	0.439 ± 0.128		
Expected heterozygosity (H_E_)	0.473 ± 0.070	0.445 ± 0.075	0.477 ± 0.055	0.583 ± 0.062	0.295 ± 0.090	0.364 ± 0.090	0.403 ± 0.102		

Mean value ± standard error. The measures are in cm, except TEET and TEE10, which are counts. The letters are paired comparison. The last columns show eigenvectors of the first (PC1) and second (PC2) principal components according to PCA

The expected heterozygosity (*H*_*E*_) levels ranged from 0.117 to 0.827 for the ten polymorphic loci. *A. mapisaga* var. *mapisaga* exhibited the highest value of *H*_*E*_ with a value of 0.586 ± 0.063, followed by the supposed *A. americana* with an average value of 0.527 ± 0.073 and *A. salmiana* var. *salmiana* with 0.470 ± 0.075, the trends being similar at the level of varieties of *A. salmiana* var. *salmiana* and *A. mapisaga* var. *mapisaga*, and a decrease in the levels of this parameter was observed in the varieties of the varieties that we consider most likely to be *A. americana* (Table 3).

**Fig. 6:**
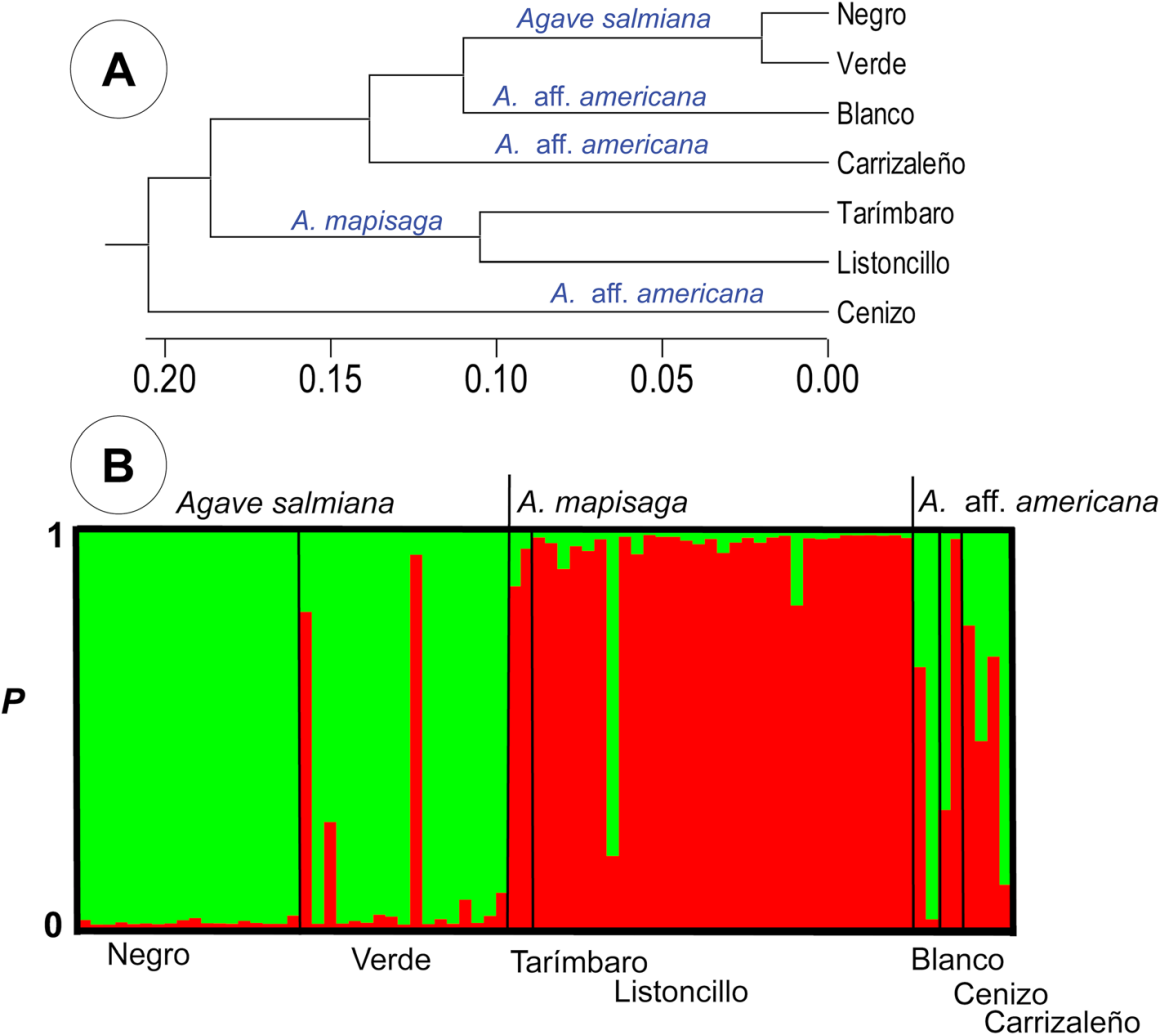
**a** Classification of varieties of *A*. *salamiana* var. *salmiana*, *A*. *mapisaga* var. *mapisaga* and most probably *A. americana* var. *americana* using Cluster analysis by UPGMA method based in Nei’s [55] genetic distance with 10 microsatellite loci. **b** Bayesian clustering in STRUCTURE, the *K* = 4 using the method described by Evanno et al., [56]. Each individual plant is represented by one vertical line with K segments coloured proportionally to their belonging to a genetic cluster

The paired genetic distances ranged from 0.0233 between the varieties “Verde” and “Negro” of the species *A. salmiana* var*. salmiana* and, 0.2088 between the varieties “Blanco” (most probably *A. americana*) and “Listoncillo” (*A. mapisaga* var. *mapisaga*). The dendrogram grouped the varieties “Verde” and “Negro” (*A. salmiana* var. *salmiana*), and varieties “Tarímbaro” and “Listoncillo” (*A. mapisaga* var*. mapisaga*) into another group. The individuals of the varieties “Blanco” and “Carrizaleño” (considered *A. americana* based on their morphology), clustered with the other varieties of *A. salmiana* var. *salmiana*, whereas the variety “Cenizo” appeared as a distinct group (Fig. 6a)*.*

The analysis of Bayesian grouping indicates that the most likely genetic groups is *K* = 2, with most individuals of the varieties of *A. salmiana* var. *salmiana* assigned to the green colour genetic group, and the varieties of *A. mapisaga* var. *mapisaga* forming the red colour group. For the three varieties considered closely related to *A. americana*, the assignment to a specific group is unclear, and individuals have similar percentages for red and green groups (Fig. 6b).

## Discussion

Results of this study indicate that there are differential management practices of agaves, as well as in the extraction of *aguamiel* and pulque production among the localities studied. The seven traditional varieties are maintained in different proportions in the production units due to their morphological characteristics and the quality of their sap. The agaves exhibit gigantism and certain characteristics related to the spines and dentition, which correspond to the features selected for their use and management. In addition, the varieties showed genetic patterns related to the degree of management and domestication as discussed in the following sections.

### Selection and maintenance of traditional varieties of *Agave*

Out of the seven traditional varieties of pulque agaves reported for both communities, only one is shared among them: “Verde” (*A. salmiana*), the most abundant variety because of the following reasons: (1) the producers of both communities perceive it as the variety that produces the best quality of sap (sweetest), (2) it produces high volumes of sap (3 L per plant per day) and (3) it has successful vegetative reproduction, producing many clones in short time.

In other regions of Mexico, a high number of traditional varieties of agave for pulque production are also reported. In Nanacamilpa and Calpulalpan, in the western region of the state of Tlaxcala, ten traditional varieties of agaves were reported by Álvarez-Ríos [60], Álvarez-Duarte et al. [61], Reyes-Agüero et al. [62], seven for *A. salmiana* and three of *A. mapisaga*. In that region, producers prefer traditional varieties of *A. salmiana* because they consider their sap to be the one with the highest quality and sweeter [60, 61]. In the *Hñähñu* region of the state of Hidalgo, Reyes-Agüero et al. [62] reported 16 traditional varieties, nine correspond to *A. salmiana* var. *salmiana*, four to *A. salmiana* subsp. *crassispina* and three to *A. mapisaga*. Also, in the latter region, *A. salmiana* var. *salmiana* is the most used specie to produce pulque, because varieties of this specie have a faster development to produce sap (6 to 8 years) than other varieties and species (e.g., 10 years for *A. mapisaga*). In addition, the traditional varieties of *A. salmiana* produce a high volume of sap, for instance, Reyes-Agüero et al. [62] reports that the largest variety “Max'o'uada” (2.7 m GPL) produce 10 L per day.

Similarly, in both localities of this study, the producers maintain other varieties of agave different from those of *A. salmiana*, and this is for different reasons. In the case of “Listoncillo” and “Tarimbaro” (*A. mapisaga*), although the sap of these varieties is of lower quality according to the perception of the producers (less sweet), these plants produce a higher volume of sap (4 L per plant per day) than those of *A. salmiana*. This is because they are varieties of greater dimensions (GPL, D, LL) and therefore with higher volumes of sap. This complementary presence of *A. mapisaga* in pulque production systems is also observed in the regions of Hidalgo and Tlaxcala, where it is common to find traditional varieties of this species, although with smaller abundances because these are less valued and used [61, 62].

In the case of the varieties “Blanco,” “Carrizaleño” and “Cenizo” (most probably *A. americana* var. *americana*), the producers maintain only few individuals of these varieties because they produce smaller volumes of sap (1.5 L per plant per day) of lower quality (less sweet to producers' perception) than the other varieties. However, with their maintenance, they are available and used when the other varieties become scarce, functioning as reserve of plant resources when the producers do not have other varieties ready to produce. The other reason for maintaining these varieties is teething (TTL, TTW, WTEE, LTEE), so these plants function as excellent living fences, delimiting and protecting terrains, crops and other agave varieties with less teething. This is an extended use in different regions of Mexico, using different *Agave* species, such as *Agave karwinskii* Zucc., *A. rhodacantha* Trel. and *A. fourcroydes* Lem, as well as those identified in this study [10, 63–65].

Producers maintain several varieties of pulque agaves because due to their properties these varieties have particular roles. The multipurpose use of agaves has been described in other rural systems in Mexico. For instance, Torres et al. [10] reported 22 categories of use for this genus, and in the systems where agaves are found, they have a structural, ecological-functional and utilitarian roles, satisfying multiple needs.

### Morphological-genetic variation and domestication of *Agave*

The identification of varieties in the studied communities accounts for the in situ maintenance of the diversity existing in this group of plants, as well as the importance of these for the communities. The traditional varieties most commonly used correspond to *A. salmiana* var. *salmiana* and *A. mapisaga* var. *mapisaga*, which are the largest sized varieties producing greater volume and sweeter sap, traits considered expressions of the domestication syndromes in the genus. Similar patterns were registered by Alfaro et al. [45] and by Mora-López et al. [36] for varieties of *A. mapisaga* the main agave crop used for preparing pulque, together with *A. salmiana* var. *salmiana* which has high morphological variation. In counterpart, the less used varieties are agaves identified most probably as *Agave americana*, which have smaller size, lower quantity and quality of their sap and whose morphological features make it difficult to handle them.

It is still necessary to conduct studies about the sweetness of the sap, to corroborate if the species and varieties mostly used produce sap with a higher content of sugars than those less used taxa. Also, more studies are needed to corroborate that saponins decrease as part of the domestication syndromes.

The cross amplification of the 11 nuclear microsatellites was viable for the species and varieties studied in this research. We find levels of genetic diversity that are in the registered range for other agaves, with the same molecular marker and similar life history characteristics. These are the cases of *Agave utahensis* subsp. *utahensis* Engelm. (*H*_*E*_ = 0.4905) and *A. utahensis* subsp. *kaibabensis* (*H*_*E*_ = 0.4081), distributed in the Mojave Desert and the Colorado Plateau in the Southwestern United States, which reproduce asexually [66]. In *A. hookeri*, a species used in the Purhépecha Plateau region for producing pulque, we reported values of genetic variation close to those reported in this study (*H*_*E*_ = 0.485), besides the fixation of heterozygotes [32], as a characteristic that distinguishes species under artificial selection, where some individuals (varieties or morphotypes) could show advantages over other genotypes [32, 35].

For the three species studied in Michoacán, the way of propagation is mainly asexual; however, the values were high compared to those registered for other species used to produce pulque, but with different molecular marker. Microsatellites are codominant and polymorphic molecular markers, and the sample size in this study is relatively small, and these could be the reason why we found high levels of genetic diversity. In fact, the results found for the putative *Agave americana* may be due to possible hybridization events, which has been mentioned as a common process in the genus *Agave* [67]. We do not rule out this possibility because eventually some individuals may “scape” from being used and having sexual reproduction; nevertheless, the possible hybrids should be still corroborated through experimental crosses. It is also pertinent to perform analyzes with conserved genome regions that would allow to establish the relationships between these varieties.

In this sense, it is important to emphasize that the exclusively vegetative propagation can lead to dramatic cases such as the “Blue agave” (*A. tequilana* var*. azul*), which due to the tequila-mezcal boom of the last decades, has led to its very low genetic diversity as a consequence of vegetative propagation through suckers. Trejo et al. [44] reported a diversity of zero in the intensive plantations of this agave in Tequila, Jalisco. Although the vegetative propagation allows an exponential increase in the size of the agave crops, taken to the extreme, it can generate problems in the genetic diversity of the populations, since it has a very limited range to generate genetic variability, which results in an offspring of genetically identical individuals, making them vulnerable to changes in weather conditions or the incidence of more aggressive pests [68].

In the studied communities, the inclusion of different varieties of pulque agaves inside a parcel, fence or patch may favour the reduction of the risk due to pests or diseases, associated with the reduction of genetic diversity in populations propagated by asexual methods.

## Conclusions

The handling of pulque in Michoacán is a current activity with cultural and economic importance to people interviewed. Local households directly consume maize, beans and fodder they produce. Livestock (cows or sheep) are used as meat during feasts or are commercialized for attending emergencies. Producing pulque offers direct benefits through its direct consumption and as a source of economic income to the families that sell the product. Everyday people sell pulque, on average 30 L per day that is 600 Mexican pesos, nearly 30 US dollars.

The most used varieties showed gigantism of the whole plant and reduction of dentition and spikiness; these tendencies are apparently result of the human selection favouring plants with these characters in greater frequency. However, the differences in the observed characteristics could be partly due to the intrinsic characteristics of each species, not only human selection. It would be recommendable, for instance, to make comparisons with the closest wild relatives of the Salmianae group (e.g. *A. salmiana* ssp. *crassispina* or *A. macroculmis*), to a better understanding of tendencies of the domestication syndrome in these species. This analysis is difficult for *A. mapisaga* since its wild ancestors remain uncertain.

Despite the vegetative reproduction of the species studied, the levels of genetic diversity were high, which may be the result of intrinsic characteristics of the species, and this genetic diversity is maintained and promoted by the management practices of maguey producers, which consist in introducing plants from other areas, which, together with the eventual sexual reproduction, enrich and maintain the diverse genetic pool in their productive systems. Although pulque producers distinguish several varieties, our morphometric and genetic studies allow identifying that *Agave salmiana* and *A. mapisaga* remain as discrete taxonomic units (species). For the varieties that were assigned to *A*. *americana*, the taxonomic identity is not clear enough and the morphology separates them as a discrete group, but the genetic information keeps these varieties as a diffuse group.

A detailed study of other phenotypic characteristics and regions of the genome that are under selection would allow to appreciate other trends of domestication in this group of plants.

Integrating studies on management and human selection influencing variation, as well as morphological and genetic characterization of such variation is crucial for understanding the nature and correct identification of variants of *Agave* species like those documented in this study and that are common in other species and species complexes of this genus.

We consider relevant the peculiar way of elaboration of pulque in SU where the sap is boiled before the elaboration of pulque; it is necessary to carry out chemical and microbiological studies to analyze the effects of this practice on the drink. It is necessary to make further studies on pulque handling and the production system in SU, since it has unusual features compared with other pulque production systems in Mexico.

## Data Availability

Please contact author for data requests.
